# Dynamic Characteristics of Rubber Reinforced Expansive Soil (ESR) at Positive and Negative Ambient Temperatures

**DOI:** 10.3390/polym14193985

**Published:** 2022-09-23

**Authors:** Jianhang Lv, Zhongnian Yang, Wei Shi, Zhaochi Lu, Qi Zhang, Xianzhang Ling

**Affiliations:** 1 School of Civil Engineering, Qingdao University of Technology, Qingdao 266033, China; 2 School of Civil Engineering, Harbin Institute of Technology, Harbin 150001, China

**Keywords:** rubber, expansive soil, backbone curve, shear modulus, damping ratio, temperature field

## Abstract

Using tire waste rubber reinforced expansive soil (ESR) can modify its poor engineering characteristics. The damping properties of ESR at different temperatures may vary dramatically. Two kinds of rubber R_a_ (large particle size) and R_b_ (small particle size) are mixed with expansive soil according to gradient ratio. The backbone curves, dynamic shear modulus, and damping ratio of expansive soil in varying temperature fields of 20 °C, −5 °C, and −15 °C are investigated. The Hardin-Drnevich model can well fit the backbone curves of ESR specimens in various temperature fields. Dynamic triaxial results show that 5–10% R_a_ rubber can withstand higher shear stress in all temperature fields; R_b_ rubber can increase the dynamic shear modulus of expansive soil and reach the peak value with 10% rubber content. The damping ratio can be significantly improved by using 10% R_a_ rubber at room temperature, while the ESR damping ratio in a temperature field of −5 °C does not change significantly with increasing shear strain or even decreases; R_a_ increases the damping ratio of expansive soils in the temperature field of 15 °C while small particle size R_b_ decreases the damping ratio of expansive soils. The experimental results validate the effectiveness of ESR in the frozen soil area. In an engineering sense, local temperature needs to be considered to use an appropriate ESR, which can provide effective seismic isolation and damping.

## 1. Introduction

Expansive soil is a kind of disastrous soil widely distributed worldwide [1,2]. It has high expansion, collapsibility, crackability, low strength, and other unfavorable engineering characteristics. These characteristics often lead to significant engineering problems such as the inclination of the upper structure, cracking of the subgrade, and slope instability in the expansive soil area, resulting in substantial economic losses worldwide every year [3,4,5].

To improve the long-term stability of the project in the expansive soil area, chemical and physical methods are mainly used to improve the expansive soil. Chemical treatment usually uses additives (lime, cement, etc.) or electrolysis to react with the central silica expansion and contraction components of silicate minerals (montmorillonite, illite, kaolin, etc.) [6,7,8,9]. Fiber materials (including natural and synthetic fibers) are commonly used for the physical treatment of expansive soils [10,11,12]. Geosynthetics [13,14,15], expanded polystyrene (EPS) [5,16] and recycled rubber [17,18,19]. The mechanism of physical methods combines external reinforcement material with expansive soil to restrain expansion and contraction ability. Previous studies have shown that rubber improves expansive soil significantly and can reuse large stocks of waste tires worldwide, which is conducive to environmental protection [20,21,22]. The compression and recompression of expansive soil gradually increase with the increase of rubber content. Signes et al. [23] found that the expansion potential of expansive soil can be reduced by 63.1% when 25% rubber particles are added. Due to the excellent performance of rubber reinforced expansive soil (ESR), the application of this composite material in geotechnical engineering has attracted wide attention in recent years [24,25].

Using waste tire rubber to modify expansive soils has achieved excellent performance. In addition to the advantages of environmental protection and control of expansion deformation, it also has a good damping capacity. Waste tire rubber is crucial to structures subjected to dynamic loads, such as building cushions requiring vibration isolation [26]. Damping ratio λ and dynamic shear modulus G are two of the most commonly used dynamic parameters in the study of dynamic properties of soft soils and have been extensively discussed for their ability to reflect the response of geotechnical materials to dynamic loads [27,28,29]. Many studies have been carried out on the dynamic properties of rubber-modified clay, mainly in the form of rubber, and the influence of particle size and rubber content on its mechanical properties. Xia et al. [30] invest the dynamic characteristics of rubber model soil were determined by using a resonance column. The damping ratio of rubber powder was 2–3 times as high as that of rubber particle treatment, and the equal change shows under different confining pressures. Similar conclusions can be drawn from the determination of dynamic properties of rubber-modified clay by dynamic triaxial method, i.e., the damping ratio of the specimens increases with the decrease of rubber particles, and it is further proposed that the optimum amount of rubber powder is 10%. Abbaspour et al. [31] reinforced clay with rubber fibers, and the damping ratio can be increased by 62% with a 1% fiber addition. Still, it decreased with an increasing proportion damping ratio of rubber fibers. Tajdini et al. [32] pointed out that mixing a certain amount of rubber particles in clay can improve the bearing capacity of the foundation, increase the flexibility of soil and increase the internal friction angle of soil, which indicates that waste rubber reinforced clay can also play a specific role in bearing static load. Load tests were carried out on sandy soils with multi-layer spacing. The results show that rubber-soil mixture (RSM) improves damping response effectively and reduces residual displacement of rock and soil when adding sandy soils with a certain thickness of RSM layers, which further verifies the excellent engineering properties of rubber-modified soils.

The above shows the standard improvement methods for expansive soil and the effectiveness of using rubber to improve expansive soil. However, there are some gaps in previous studies. On the one hand, the optimal proportion between the shape of the recycled rubber used and the soil mix has not been determined. On the other hand, the influence of temperature change on the composite improved soil (especially negative temperature) was not considered. In the world, there are expansive soil disasters that are widely distributed [33,34]. Especially in the seasonal and perennially frozen soil areas, there are often more severe geological disasters because of the impact of water phase change [35]. There is an urgent need for research on whether ESR can be used in low-temperature areas, but the research in this area is still blank. When the rubber is in a low-temperature state, its volume, strength, and damping capacity will dramatically change [36]. Therefore, the potential of using ESR materials in permafrost regions must be evaluated. On this basis, we selected different test temperatures, different rubber forms, and different adulteration ratios to explore the potential ability of ESR, a composite material, to improve the expansive soil disasters in frozen soil regions. Through this study, we hope to provide the best rubber form and proportion for ESR improvement in expansive soil engineering in frozen soil regions.

## 2. Experimental Methodology

### 2.1. Materials

#### 2.1.1. Expansive Soil

For expansive soil research, due to its broad global distribution, the expansive potential is commonly used to classify expansive soils in processes that do not involve chemical changes. The more enormous the expansion potential, the more significant the volume change during water absorption and loss, and the more disastrous the project will be. Our research is to select a kind of medium-expansive soil that is generally representative. The improvement of this expansive soil is of new significance worldwide.

**Figure 1:**
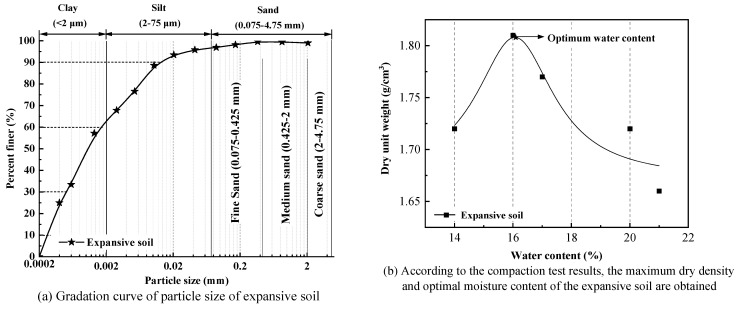
Grain-size distribution curves (**a**) and compaction test (**b**) for the soil.

The test uses the expansive soil produced in Meicun, Weifang, China. Its basic physical properties are shown in Table 1. The specific gravity of the expansive soil measured according to ASTM d854–14 (2014) [37] is 2.71; according to ASTM d4318-17e1 (2017) [38], the liquid limit is 44.7%, the plastic limit is 22.7%, and the plasticity index is 22.0% > 17.0%. Therefore, the expansive soil for test can be divided into clay. Prakash and Sridharan [39] studied the expansion and contraction characteristics of common expansive soils and classified them. It is measured that the free expansion ratio of expansive soils used in this test is 1.71, which can be divided into moderate expansive soils. Figure 1a shows the particle size grading of the test clay. It can be seen that the expansive soil is mainly composed of clay fine-grained soil. According to ASTM d698–12 (2012) [40], the maximum dry density and optimal moisture content of expansive soil are determined by a compaction test. The compaction test results are shown in Figure 1b.

#### 2.1.2. Recycled Tire Rubber

One of the research purposes of this paper is to solve the problem of waste tires in solving engineering disasters of expansive soil. Therefore, this research uses scrap automobile tire rubber, which is mainly composed of styrene butadiene rubber. The test used rubber powder produced in the Deyang tire treatment plant, Sichuan, China. According to the production instructions of the factory, the production process of recycled rubber powder is as follows:

① Removing bead wire and using a crusher to divide the rubber into 3–5 cm rubber blocks;
② Use the rubber intermediate crusher to break the rubber block into 10–20 mm rubber particles;
③ Use the rubber fine crusher to break the rubber particles into 1–3 mm or 3–6 mm rubber particles;
④ At average temperature, use a fiber sorter to separate the rubber from the nylon fiber in the tire;
⑤ Use the rubber fine rubbing machine to divide the rubber particles into 0.60–0.09 mm rubber powder.

**Figure 2:**
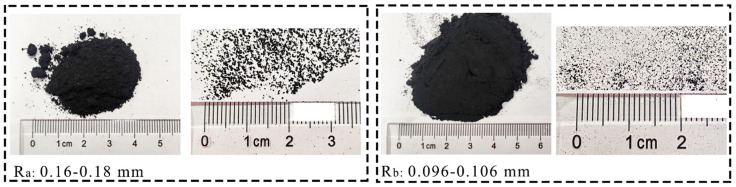
Two kinds of rubber powders R_a_ and R_b_ were selected for the test.

Rubber size has a significant impact on the ESR damping ratio. Xia et al. [30] and Akbarimehr and Fakharian [29] measured that the damping ratio of rubber powder-modified expansive soil is greater than that of rubber particle-modified soil. Therefore, recycled rubber is selected as rubber powder in this test. Figure 2 shows that two kinds of recycled rubber powders with different particle sizes were selected for the test. The first was 0.16–0.18 mm (R_a_); The second is 0.096–0.106 mm (R_b_).

### 2.2. Experiments

#### 2.2.1. ESR Specimens Preparation

**Figure 3:**
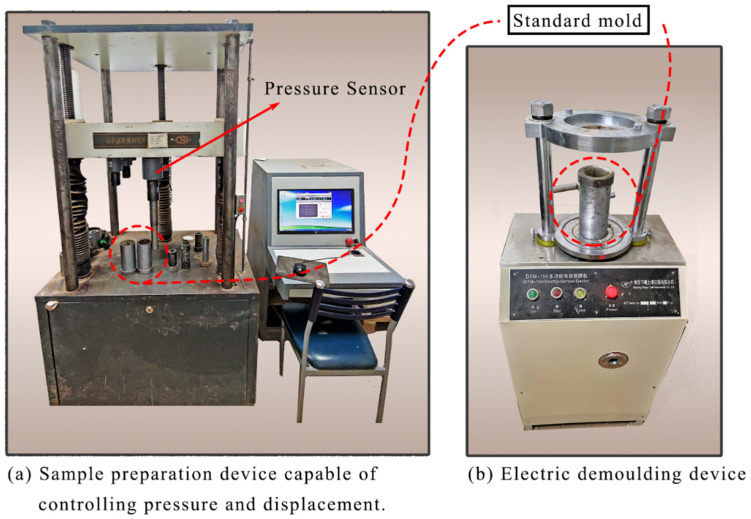
Standard specimens auto-preparation device and specimens auto-release machine. (**a**) Specimens preparation machines. (**b**) Mold release machine.

**Figure 4:**
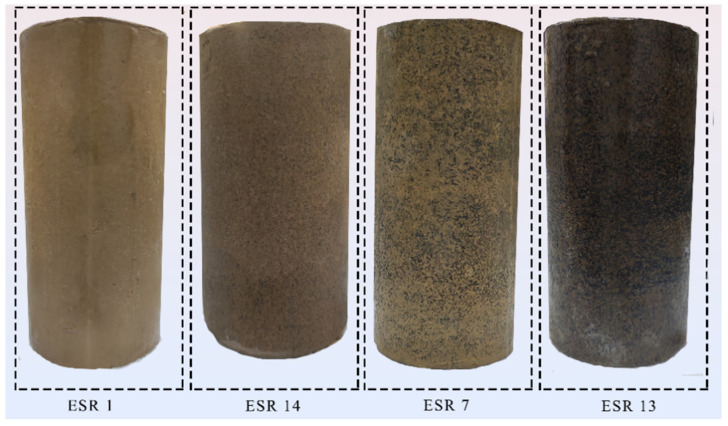
Standard specimens of different rubbery contents.

Akbarimehr et al. [41] measured the damping ratio of rubber-modified clay and proposed that the optimal rubber content is 10%. When the rubber content exceeds 30%, it is challenging to prepare uniform test specimens because of the influence of rubber. Therefore, four rubber content variables, 5, 10, 15, and 20%, were set up based on 10% rubber content. Specimens number and setting variables (rubber particle size, temperature, rubber content) are shown in Table 2. The compaction method is often used to treat expansive soil in practical projects. To ensure the engineering significance of the test results, the ESR specimens are prepared according to the optimal water content and maximum dry density of expansive soil. The specimens are prepared by mixing the dry soil powder with the rubber powder in proportion, then spraying the water corresponding to the optimal water content evenly into the soil, and then standing in an airtight container for 24 h. The above rubber content is prepared as a percentage of the total quality of the soil specimens. The ESR specimens were prepared using a specimen preparation device independently developed by the National Laboratory of Permafrost Engineering, Chinese Academy of Sciences (Figure 3a). This device monitors the pressure applied during the preparation of standard specimens and controls the drop displacement, which guarantees the uniformity of the specimens and a certain degree of compactness. ESR soil is first loaded into the molds and lightly compacted during specimen preparation. The sampling equipment then compacts each specimen to 61.8 × 125 mm standard specimens in both directions. The ESR specimens are stabilized at constant pressure for 6 h in the molds to ensure uniformity. An automatic demolding device is used for the ESR specimens (Figure 3b) to separate specimens from molds. After the preparation of specimens, a three-valve mold is used to fix specimens, saturate them in a vacuum saturated cylinder for 24 h, and measure the height of the specimens after saturation. After saturation, the cryogenic test group freezes the specimens at the test temperature for 24 h along with the mold, strictly ensuring that the ambient temperature of the specimens remains at the design value during the test. Figure 4 shows the partially prepared ESR specimens, with rubber content increasing from left to right.

#### 2.2.2. Dynamic Triaxial Test

**Figure 5:**
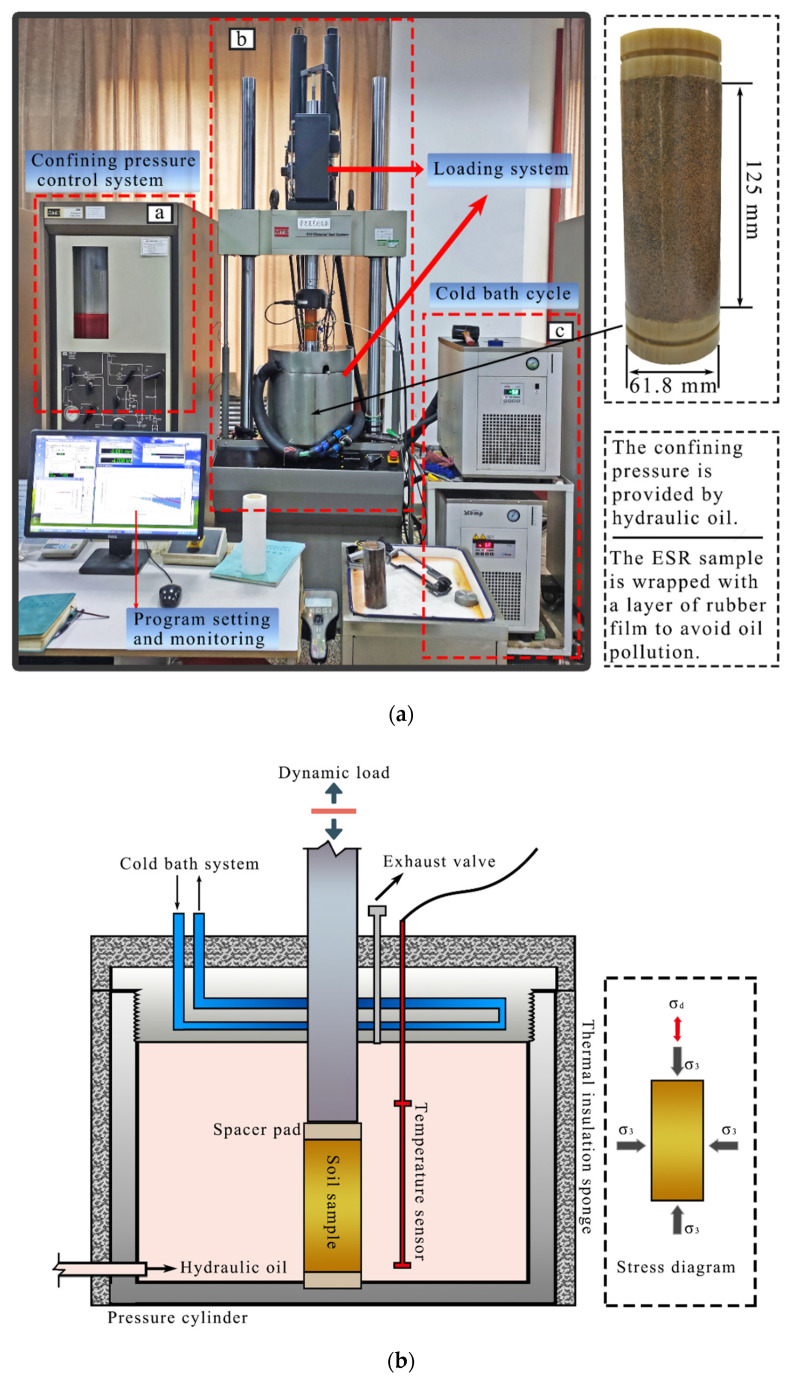
Dynamic triaxial machine (MTS-810(5T)) and specimens. (**a**) The motor triaxial testing machine contains three parts: a. The perioperative pressure control system; b. Loading system; c. Cold bath circulatory system. (**b**) hydraulic oil provides ambient pressure and temperature control is controlled by the cold bath unit and the upper and lower temperature sensors; air should be exhausted by the exhaust valve before testing.

As shown in Table 2, the confining pressure of all tests during dynamic triaxial cyclic loading is 0.4 MPa, to ensure the practical engineering value of a dynamic triaxial test. Figure 5a shows MTS–810 (5T) in the National Laboratory of Permafrost Engineering, Chinese Academy of Sciences. Dynamic triaxial tester, with maximum axial load: 5 T; maximum axial deformation: +75 mm; enclosure range: 0.3–10 MPa; frequency range: 0–50 Hz; temperature range: 20–−20 °C. The dynamic triaxial tester consists of three parts, shown in Figure 5a, a- Control system of enclosure pressure; b-Loading system; c-Cold bath circulation system. The enclosure pressure, loading load and axial force, displacement and enclosure pressure during the test can be set by a computer or monitored in real-time. Cold bath circulation can keep the temperature in the cold bath tube constant. All specimens are tested after consolidation, and the temperature remains constant for 30 min. All test results are completed after consolidation under undrained conditions (CU) for the ESR frame to bear stress directly. Figure 5b is a dynamic triaxial loading diagram. Air should be discharged before the test, waiting for the hydraulic oil temperature to reach the preset value and starting the test after the specimens have been consolidated.

**Figure 6:**
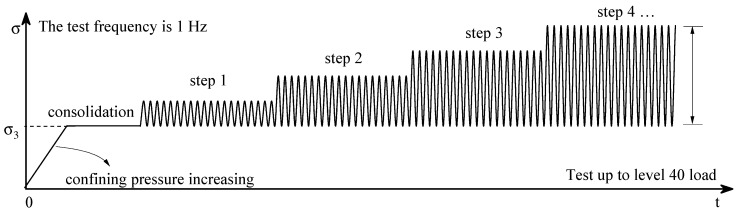
Test load.

The test uses sinusoidal dynamic loads at a frequency of 1 Hz. The design consolidation ratio is 1. As a subgrade, the upper structure will be deformed and destroyed by vibration stress during the earthquake. As an expected seismic isolation material, it is necessary to test the dynamic characteristics of ESR to verify its performance in the earthquake. The test is carried out under 0.4 MPa for a multi-stage dynamic load test, with a total of 40 dynamic loads designed and each stage cycling 40 times. The first 20 dynamic loads stabilize the soil mass, and the last 20 test data are used to test the dynamic parameters. As shown in Figure 6, the dynamic load increases in a step-by-step mode with an initial load of a smaller dynamic load which increases in equal magnitude. The failure criteria used in the tests are elastic and plastic strains and transient stress changes of up to 5%. To explore the dynamic characteristics of ESR, the test selected the two winter characteristic temperatures in Yanji Expansive Soil Area, Jilin Province, China [42]. Low-temperature preset temperature: −5 °C and −15 °C, and 20 °C as positive temperature preset temperature. All test variables are shown in Table 2.

### 2.3. Description of The Dynamic Model of Frozen Soil for Fitting

Under dynamic load, soil shear modulus and damping ratio are often considered. The former represents the bearing capacity of soil under dynamic load, while the latter represents the energy dissipation capacity of soil during vibration. According to classical dynamics theory, the defined process can be described as follows.

**Figure 7:**
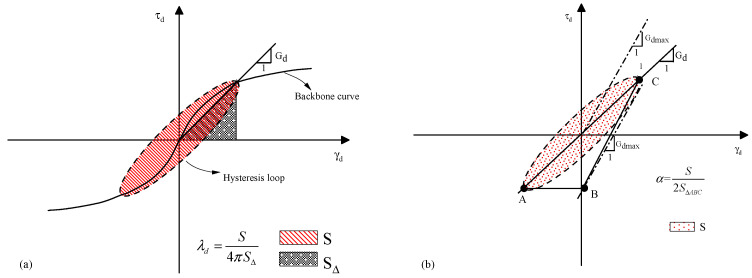
Geometric illustration of dynamic shear modulus and damping ratio. (**a**) Backbone curve and damping ratio calculation. (**b**) Dynamic shear modulus schematic.

Dynamic stress $\sigma_{d}$ and dynamic strain $\varepsilon_{d}$ can be obtained in a dynamic triaxial test. According to the basic formulas of material mechanics, Equations (1) and (2), shear stress and shear strain can be obtained. Equation (3) is the formula for calculating the dynamic shear modulus, defined as the ratio of shear stress to shear strain, i.e., the backbone curve slope shown in Figure 7.

(1) $$\gamma_{d}=\varepsilon_{d}(1+\mu )$$

(2) $$\tau_{d}=\sigma_{d}/2$$

(3) $$G_{d}=\tau_{d}/\gamma_{d}$$

where μ is the Poisson ratio of the material, which can be considered constant at 0.5 for a saturated clay and does not vary with the peri- and shear strain [43]. Hardin and Drnevich [44,45] proposed the classic hyperbolic versus backbone curve fitting equation Equation (4) for sandy soil versus clay, and numerous scholars have verified its accuracy in matching the dynamic shear moduli of sandy soil versus clay [46,47,48].

(4) $$\tau_{d}=\frac{\gamma_{d}}{a+b\gamma_{d}}$$

where *a* and *b* are the fit parameters for the trial, a>0,b>0. According to Hardin and Drnevich [44,45], the damping ratio can be formulated as:

(5) $$\lambda_{d}=\frac{S}{4\pi S_{\Delta }}$$

where S and $S_{\Delta }$ defined as in Figure 7a. Zhao et al. [49] state that the damping ratio can be more simply expressed as:

(6) $$G_{d\max}=\underset{\gamma_{d}\to 0}{\lim}G_{d}=\frac{1}{a}$$

(7) $$\tau_{dult}=\underset{\gamma_{d}\to +\infty }{\lim}\tau_{d}=\frac{1}{b}$$

(8) $$\gamma_{dr}=\frac{\tau_{dult}}{G_{d\max}}=\frac{a}{b}$$

(9) $$\lambda_{d}=\frac{2\alpha }{\pi }\left(1-\frac{G_{d}}{G_{d\max}}\right)=\lambda_{\max}\left(1-\frac{G_{d}}{G_{d\max}}\right)=\lambda_{\max}\left(\frac{\gamma_{d}/\gamma_{dr}}{1+\gamma_{d}/\gamma_{dr}}\right)$$

where α is a constant, is the ratio of half the shaded area to the triangular ABC area in Figure 7b and is the maximum damping ratio.

## 3. Results and Discussion

### 3.1. Backbone Curve of ESP at Different Temperatures with Different Rubber Forms and Dosage

**Figure 8:**
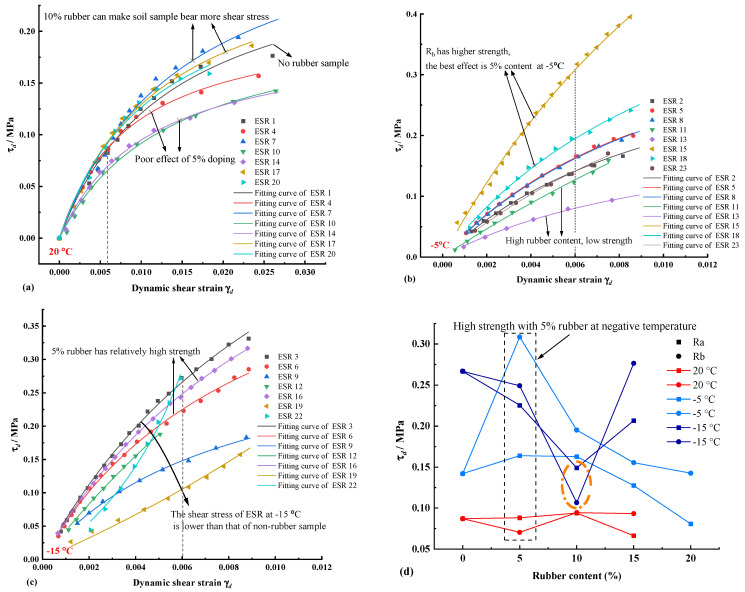
Backbone curve and upper shear stress limit of expansive soil under different rubber forms and yields. (**a**) 20 °C ESR specimens. (**b**) −5 °C ESR specimens. (**c**) −15 °C ESR specimens. (**d**) Upper shear stress limit varies with rubber content.

The ESR backbone curves of different rubbery forms incorporated at three temperatures were considered in Figure 8. Figure 8a described various kinds of ESR at room temperature in contrast to the use of rubber-modified test specimens, it can be found that ESR 7, ESR 17 specimens, that is, expansive soil at 10% rubber incorporation, can bear more enormous shear stress at room temperature 20 °C. Comparing the two rubber powders R_a_ with R_b_, for the expanded clay with smaller particle size, R_a_(with larger particle size) has a better modification effect, and only 10% rubber contained corresponds to higher stress in the backbone curve than in the unmodified specimen. However, Xia et al. [30] suggested that a smaller modified rubber particle size is better for the use of expansive soil for this phenomenon, possibly since the incorporation of rubber particles with a larger particle size can refine the gradation of a fine-grained clay, thus optimizing the expansive soil framework and effectively improving the load-bearing capacity of the mass. Xia et al. [30] used larger soil particles, producing better results using finer rubbers. Akbarimehr and Fakharian [29] conducted a static triaxial test at room temperature on clay modified with rubber powder and found that the use of clay modified with different rubber powders resulted in various degrees of load-bearing loss, mainly due to the smaller strength of the rubber material than the clay. The above phenomenon is also well explained in Figure 8a. Most ESR specimens had less shear stress than those without rubber treatment, i.e., specimens ESR 4 and ESR 14 at 5% rubber content withstood less shear stress than ESR 1 (no added rubber soil).

Figure 8b,c depicts the backbone curve changes of ESR below 0 °C (−5 °C and −15 °C), and there is a clear difference in the ESR shear stress upper bounds between the two states. When at −5 °C, the ESR 5, ESR 8, ESR 15, and ESR 18 specimens with 5% and 10% rubber content had a noticeable increase in the upper shear stress limit corresponding to the same shear stress. In contrast, the ESR 11, ESR 13, and ESR 23 with more than 10% rubber content had an evident decrease in the upper shear stress limit compared to ESR 2. The above phenomena illustrated a higher upper limit of shear stress at the temperature of −5 °C in the rubber-modified expanded specimens with 5–10% content. R_b_ with a finer grain size was significantly promoted compared to R_a_ shear stress.

It is noteworthy that the upper shear stress limit of ESR15 is significantly higher than that of other specimens. Comprehensive test data show that this is since 5% of R_b_ absorbs more water when saturated under the same conditions, which leads to more ice crystals in the specimens during the negative temperature test and increases the upper shear stress limit of the specimens. It can be found from Figure 8c that the upper shear stress limit of all ESR specimens is lower than that of ESR3 without rubber added when the temperature is −15 °C. Similarly, at −15 °C, a relatively high upper shear stress limit still exists for 5% rubber content. From the backbone curves of ESR specimens at two negative temperatures, it can be found that most of the free water in the expansive soil is unfrozen, and the unfrozen water content is relatively large when the temperature is just below 0 °C. At this time, rubber particles can increase the internal friction angle of the soil and, thus, the upper shear stress limit. The strength of frozen expansive soil increases gradually as the temperature of the specimen decreases to −15 °C. Still, the strength of rubber increases limited with the temperature at 0 °C, resulting in lower shear stress of ESR.

The backbone curves are fitted by the Hardin-Drnevich model (Equation (4)). All fitting parameters are shown in Table 3 and the fitting effect is good. This indicates that the H-D model can reasonably predict the backbone curve of ESR at positive and negative temperatures. Figure 8d analyzed the change of ESR rubber content and the change of upper shear stress limit at different temperatures under the same strain and found that the shear stress is significantly greater than that at room temperature when the temperature is lower than 0 °C. The shear stress of R_b_ with smaller particle size is higher in most cases, and the rubber with 5% content generally has a better improvement effect at negative temperatures. Still, the upper shear stress limit gradually decreases with the increase of rubber content. It is noteworthy that when the temperature is −15 °C, the shear stress of R_a_ and R_b_ in both rubber specimens has a minimum value of 10%. This indicates that at lower temperatures, the upper limit of ESR shear stress will increase first and then decrease progressively with the increase of rubber content, i.e., the strength of the specimen will change from expansive soil control to rubber control, and the upper limit of shear stress will increase.

### 3.2. Dynamic Shear Modulus of ESP at Different Temperatures with Different Rubber Forms and Dosage

**Figure 9:**
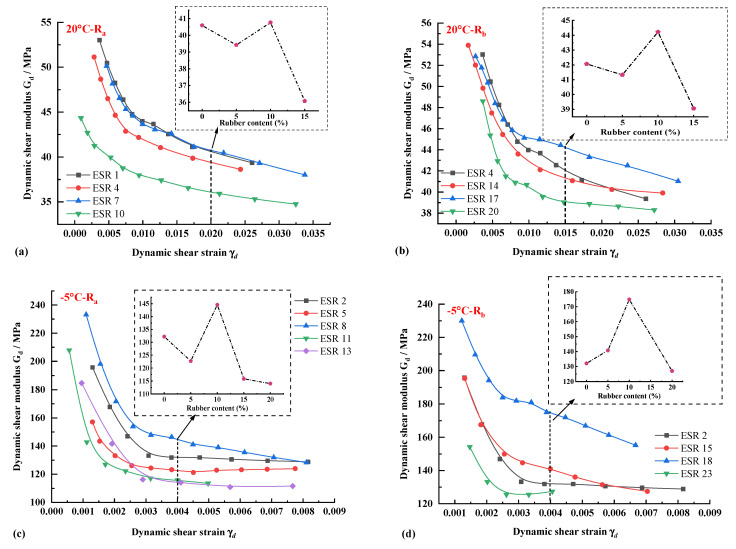

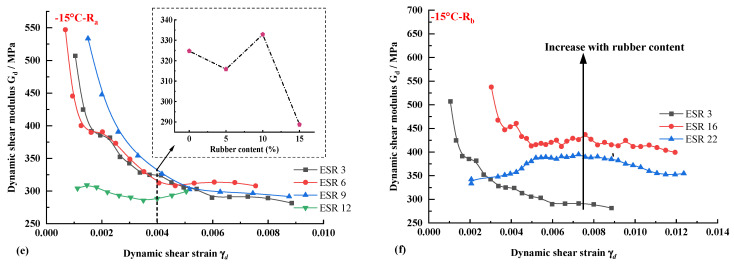
Dynamic shear modulus of expansive soil under different rubber forms and yields. (**a**) 20 °C R_a_ specimens. (**b**) 20 °C R_b_ specimens. (**c**) −5 °C R_a_ specimens. (**d**) −5 °C R_b_ specimens. (**e**) −15 °C R_a_ specimens. (**f**) −15 °C R_b_ specimens.

Figure 9 describes the dynamic shear modulus changes of ESR specimens at different temperatures. Figure 9a,b is the change of shear modulus of two kinds of rubber at room temperature at 20 °C. It can be found that the dynamic shear modulus of R_a_ specimens with relatively large particle sizes does not increase significantly. In contrast, the dynamic shear modulus of ESR17 specimens with 10% R_b_ addition increases significantly. The changing trend of the shear modulus of the two rubber-reinforced specimens is the same at 20 °C. The shear modulus decreases at 5% rubber content and increases at 10% rubber content. Then the dynamic shear modulus decreases again with the increase of rubber content. This phenomenon is mainly because rubber particles can make the expansive soil particles more compact under stress and increase the contact area between soil particles. In other words, when subjected to dynamic load, it is divided into loading and unloading stages. In the loading stage, rubber particles can produce large deformation, which benefits the contact and direct transmission of soil particles in the soil frame. When unloaded, the rubber material can quickly restore its original state and provide specific elastic force.

The dynamic shear modulus changes differently when ESR specimens are at low temperatures of −15 °C. There is a significant difference between the rubber R_a_ and R_b_. As shown in Figure 9e,f, R_a_ doping has no significant effect on the dynamic shear modulus. Still there is a substantial increase in ESR 16 and ESR 22 of R_b_ specimens because smaller particle sizes of R_b_ can significantly reduce the porosity in expansive soils and improve the bearing capacity of soils. Rubber, as a sound damping material, also has a significant influence on the damping ratio of soils, which will be discussed in Section 3.3. In combination with the above analysis, it can be seen that the dynamic shear modulus of expansive soil can be significantly increased when 10% rubber powder is added in different temperature fields, and a better optimization effect can be achieved when using small particle size R_b_, which is more significant in the temperature field of −15 °C at lower temperatures.

### 3.3. Damping Ratio of ESP at Different Temperatures with Different Rubber Forms and Dosage

**Figure 10:**
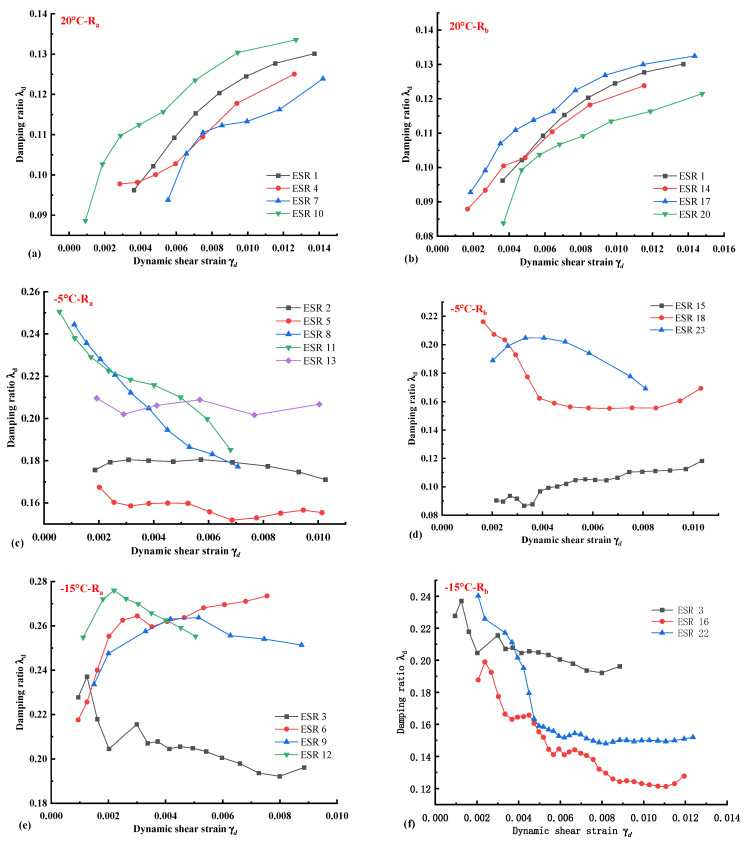
Damping ratio of expansive soil under different rubber forms and yields. (**a**) 20 °C R_a_ specimens. (**b**) 20 °C R_b_ specimens. (**c**) −5 °C R_a_ specimens. (**d**) −5 °C R_b_ specimens. (**e**) −15 °C R_a_ specimens. (**f**) −15 °C R_b_ specimens.

Rubber has always been considered a sound damping material and is often used in building vibration isolation design. However, as a temperature-sensitive material, there are temperature deformations and changes in the damping capacity of rubber material in different temperature fields, so differences in ESR damping ratio at different temperatures need to be studied. Figure 10 shows the damping ratios of different ESR specimens in three temperature fields. The development trend of damping ratios in various temperature fields is significantly different. At room temperature, the damping ratio of expansive soils in Figure 10a,b changes gradually with increasing strain. When R_a_ is added to 15%– ESR 10 and R_b_ is added to 10%–ESR 17. The damping ratio is more extensive than ESR 1 without rubber, which has a better damping energy absorption effect.

The damping ratio of ESR specimens varies significantly with strain when they are in the low-temperature fields of −5 °C and −15 °C. The damping ratio of ESR specimens doped with rubber increases significantly when Figure 10c,d is −5 °C. However, the damping ratio does not change significantly with the increase of strain at low temperature and even decreases with the increase of strain (ESR 8, ESR 11, ESR 16, and ESR 22). The damping ratio of expansive soils can be increased significantly by adding a large particle size R_a_ in the temperature field of −5 °C. Overall, with the increase in rubber content, the damping ratio also increases. Still, the influence of different rubber content on the damping ratio is not apparent, and the damping ratio curve corresponds to various contents changes within a specific range.

However, in the temperature field Figure 10e,f of −15 °C at a lower temperature, the optimum damping ratio of the two rubber types is quite different. Compared with ESR 3 without rubber added, the damping ratios of three R_a_ specimens are significantly increased, but small particle size R_b_ decreases the damping ratios of expansive soils. This is because small-size rubber has minor deformation in the expansive soil, and it is challenging to dampen the deformation of the rubber itself. At the same time, the failure of expansive soil under negative temperatures presents plastic characteristics. During the process of bearing dynamic load, there are micro-cracks in the soil, which gradually develop. The existence of micro-cracks further makes the damping effect of small-size rubber challenging to play a role. In addition, the expansive soil has water loss shrinkage characteristics. The freezing process is the process of water loss of the expansive soil. The water loss shrinkage of the expansive soil will cause the contact between rubber and soil particles to become less tight. As a result, the damping effect of expansive soils is reduced instead of increasing the damping ratio of small-size rubber powders. Wei et al. [50] also drew similar conclusions in their study on the dynamic properties of rubber-modified clay during freeze-thaw cycles. Wei et al. [50] believed that material differences between the modified clay and soil would lead to structural instability under different temperatures, which in turn would affect the dynamic properties of rubber-modified clay. Therefore, the fitting effect of the ESR damping ratio of expansive soil and increased rubber is worse than that of the specimen using Equation (9). The damping ratio gradually increases with strain at different temperatures and changes significantly in the smaller strain range. It is difficult to find a suitable fitting curve in the smaller strain range, which needs further study.

## 4. Conclusions

In this study, two kinds of rubber powders with different particle sizes were added to expansive soil according to the gradient content, and it was improved to ESR. A series of dynamic triaxial tests were carried out to determine the dynamic characteristics of ESR specimens. The test was carried out in three temperature fields, including one positive temperature of 20 °C and two negative temperatures of −5 °C and −15 °C. the backbone curves, dynamic shear modulus, and damping ratio of ESR specimens under different temperature fields were measured. The test shows that the dynamic characteristics of expansive soil at different temperatures can be significantly improved by using reasonable rubber particle size and content. The results are as follows:

(1) The backbone curve of ESR specimens under different temperature fields can be better fitted by the H-D model. 10% rubber content at room temperature can improve the upper limit of specimen shear stress in the backbone curve. 80 mesh R_a_ rubber with a large particle size can optimize the grading curve of expansive soil so that the soil skeleton of expansive soil can bear tremendous shear stress. At −5 °C, 5–10% rubber can increase the upper limit of shear stress of expansive soil, and the effect of 150 mesh R_b_ rubber with a smaller particle size is better. At the same time, the higher the moisture content in the specimen, the greater the upper limit of shear stress in the backbone curve.

(2) At room temperature, small particle size R_b_ can significantly improve the dynamic shear modulus of expansive soil, and the dynamic shear modulus of all specimens increases further with the decrease of temperature. Under all temperature fields, the dynamic shear modulus has the same trend as the rubber content, which increases to 10% to reach the peak, and then decreases gradually. When the temperature field is −15 °C, the dynamic shear modulus of R_b_–ESR specimen is higher than that of the plain soil specimen.

(3) In each temperature field, 10% R_a_ rubber can significantly improve the damping ratio of expansive soil. The negative temperature state of expansive soil will lose water and shrink, resulting in the loose soil skeleton due to the loose contact between soil and rubber. At a lower temperature of −15 °C, R_b_ rubber powder with a smaller particle size will reduce the damping ratio of expansive soil. The damping ratio of expansive soil and ESR changes sharply with shear strain in low-temperature field, so it is difficult to fit with the formula of damping ratio of low-temperature clay.

(4) In case of expansive soil disaster in frozen soil engineering, using the appropriate proportion of rubber ESR can effectively improve the dynamic characteristics of expansive soil. The use of 80 mesh R_a_ and 10% can effectively improve the upper limit of shear stress, dynamic shear modulus, and damping ratio of backbone curve in different positive and negative temperature fields, which can play a better engineering effect. However, the dynamic response of ESR in a large-scale model still needs to further study. When ESR is used to improve the dynamic characteristics of subgrade below 0 °C, the improper rubber form and content will reduce the dynamic characteristics of expansive soil.

## Figures and Tables

**Table 1:** Physical properties of the selected expansive soil.

Properties	Values	Standard Designation
$\mathrm{Specific}\;\mathrm{gravity},\;G_{s}$	2.7	ASTM D854-14
Natural moisture content (%)	5.7	ASTM D2216-19
Free expansion ratio	1.7	Prakash and Sridharan (2004)
$\mathrm{Liquid}\;\mathrm{limit},\;\omega_{L}$ (%)	44.7	ASTM D4318-17e1
$\mathrm{Plastic}\;\mathrm{limit},\;\omega_{P}$ (%)	22.7	
$\mathrm{Plasticity}\;\mathrm{index},\;I_{p}=\omega_{L}-\omega_{P}$ (%)	22	
$\mathrm{Optimum}\;\mathrm{water}\;\mathrm{content},\;\omega_{opt}$ (%)	16.1	ASTM D698-12
$\mathrm{Maximum}\;\mathrm{dry}\;\mathrm{unit}\;\mathrm{weight}\;\gamma_{d\max}$ (g/cm^3^)	1.8	

**Table 2:** Specimens’ parameters for ESR test.

Specimens Number	Rubber Type	Rubber Content (%)	Test Temperature (°C)	Confining Pressure (MPa)
ESR1, ESR2, ESR3	−	0	20, −5, −15	0.4
ESR4, ESR5, ESR6	R_a_	5	20, −5, −15	
ESR7, ESR8, ESR9	R_a_	10	20, −5, −15	
ESR10, ESR11, ESR12	R_a_	15	20, −5, −15	
ESR13	R_a_	20	−5	
ESR14, ESR15, ESR16	R_b_	5	20, −5, −15	
ESR17, ESR18, ESR19	R_b_	10	20, −5, −15	
ESR20, ESR21, ESR22	R_b_	15	20, −5, −15	
ESR23	R_b_	20	−5	

**Table 3:** Regression analysis constants a and b for Equation (4)—H-D model.

Specimen Number	a	b	R^2^	Specimen Number	a	b	R^2^	Specimen Number	a	b	R^2^
ESR1	0.04791	3.49434	0.98582	ESR9	0.02303	2.91256	0.99857	ESR17	0.04293	3.44147	0.99477
ESR2	0.02758	2.46981	0.9975	ESR10	0.06292	4.60505	0.99537	ESR18	0.02101	1.6325	0.99739
ESR3	0.01542	1.19097	0.99784	ESR11	0.04118	1.00078	0.99691	ESR19	0.06447	1.25043	0.99472
ESR4	0.0401	4.6462	0.99584	ESR12	0.02233	0.85672	0.99855	ESR20	0.04238	3.66478	0.98905
ESR5	0.02411	2.10156	0.99838	ESR13	0.04907	4.28004	0.99942	ESR21	0.04549	2.15476	0.97982
ESR6	0.01615	1.75391	0.99793	ESR14	0.05519	5.01866	0.99704	ESR22	0.04358	3.74433	0.99299
ESR7	0.04577	3.01046	0.98937	ESR15	0.01478	0.78268	0.99889	ESR23	0.03173	1.77198	0.99756
ESR8	0.02461	2.07235	0.99942	ESR16	0.01607	1.34628	0.99981				

## Data Availability

Not applicable.
